# Threonine sulfation: a rare post translational modification in insect adipokinetic hormones

**DOI:** 10.1038/s41598-026-50205-x

**Published:** 2026-05-15

**Authors:** Gerd Gäde, Simone König, Sameer S. Kulkarni, Richard J. Payne, Heather G. Marco

**Affiliations:** 1 Department of Biological Sciences, University of Cape Town, Rondebosch, South Africa; 2 Service Unit Proteomics, Medical Faculty, University of Münster, Münster, Germany; 3 School of Chemistry, The University of Sydney, Sydney, NSW 2006 Australia; 4 Australian Research Council Centre of Excellence for Innovations in Peptide and Protein Science, The University of Sydney, Sydney, NSW 2006 Australia

**Keywords:** Post-translational modifications, Adipokinetic hormone, Insect, Threonine sulfation, Mass spectrometry, Solid-phase peptide synthesis, Biochemistry, Physiology, Zoology

## Abstract

Peptides of the adipokinetic hormone family are responsible for metabolic roles in insects, regulating release of energy metabolites from the fat body. We report on adipokinetic hormone octapeptide sequences bearing a rarely identified post-translational modification (sulfation of a threonine residue) in two beetle subfamilies (Cetoniinae and Dynastinae) of the large superfamily of Scarabaeoidea (dung beetles, rhinoceros beetles and flower beetles), and in a bug species (family Coreidae). In the cetonids *Pachnoda sinuata*,* Dicronorhina derbyana derbyana*,* Tropinota hirta*,* Protaetia cuprea*,* Cetonia aurata* and *Oxytherea funesta* sulfated Pacsi-AKH is found (pQINLTsTGW amide), while sulfated Penid-AKH (pQVNISsTGW amide) occurs in the dynastid beetles *Pentodon idiota*, *Xylotrupes gideon* and *Syrichthodontus spurius.* Sulfated Schgr-AKH-II (pQLNFSsTGW amide) is found in the twig wilter *Holopterna alata*. Sequence elucidation was achieved by mass spectrometry, however, due to the labile nature of the sulfate group under mass spectrometric conditions, the modified amino acid could not be easily identified. Edman degradation and comparative mass spectrometry evaluations with synthetic sulfopeptide standards were therefore employed for sequence validation. This type of sulfation was previously only reported present on the protein backbone of very few proteins from vertebrates (including humans), a mollusc and a protozoan parasite.

**Supplementary Information:**

The online version contains supplementary material available at 10.1038/s41598-026-50205-x.

## Introduction

The adipokinetic hormone (AKH) peptide family is one of the most studied hormone families in insects. It is part of the gonadotropin-releasing hormone superfamily^1–5^ and its main function in insects is in intermediary energy metabolism, viz. being responsible for the release of diacylglycerols and/or trehalose from the fat body during extensive locomotory activity^6–8^. These peptides are exclusively synthesised in neurons in the retrocerebral corpora cardiaca (CC) glands. Structurally, the AKH family is characterised by a peptide length of 8 to 10 amino acids with at least two aromatic acids, Phe or Tyr, at position 4 from the N-terminus and the signature amino acid Trp at position 8. Typically, position 2 features an aliphatic Leu, Ile or Val residue, although an aromatic amino acid (Phe) has also occupied this position in some cases. Other features include Asn or Thr at position 3, Thr or Ser at position 5 and post-translational modifications (PTMs) which are introduced at the termini – pyroglutamatic acid (pGlu) at the N-terminus and a carboxyamide at the C-terminus^9,10^. In contrast to other insect peptide families, the AKH family has been shown to have a range of further and sometimes rare PTMs. For example, a decapeptide of a stick insect was found with a C-mannosylated Trp residue^11–13^, while a possible isomerisation of Pro occurs in a cicada decapeptide^14^. Proline hydroxylation has been detected in AKHs of various species^1,15–17^; Thr phosphorylation is observed in a beetle species^18^, while a conference communication reported Thr sulfation in a single bug species^19^. The current study is a detailed report showing that Thr sulfation of the AKH peptide extends also to beetles.

Amongst all the proteinogenic amino acids, tyrosine remains the most widely known amino acid to feature O-sulfation. Chemically this process involves transfer of an inorganic sulfate from 3′-phosphoadenosine-5′-phosphosulfate (PAPS) onto the phenolic side-chain of a tyrosine residue to generate sulfated tyrosine (sulfoTyr), with this process catalysed by an enzyme from one of two tyrosylprotein sulfotransferases (TPSTs)^20,21^. The first described sulfoTyr containing peptide was derived from bovine fibrinogen^22^. Subsequently, many more sulfoTyr containing peptides have been chemically elucidated including peptide hormones of vertebrates such as gastrin and cholecystokinine (CCK)^23,24^, sulfopeptides derived from hematophagous organisms such as leeches, ticks and mosquitoes^25,26^, and other insects (e.g. sulfakinins) to name a few^27,28^. Recently, this modification also became the object of studies in plants where plant hormones such as phytosulfokines, root meristem growth factors and Casparian strip integrity factors play a decisive role in growth, development and reproduction^29^.

But what about sulfation of other amino acids? While in 2004 a few proteins from a snail (*Lymnaea stagnalis*), a Malaria parasite (*Plasmodium falciparum*) and humans were reported to possess sulfoThr (also denoted as sT or sThr in the current study) and sulfoSer modifications^30^, no peptide has been shown to have a sulfoThr incorporated, to the best of our knowledge. Only recently, the primary structure of a unique sulfated molecule was elucidated from a unicellular silica alga (the diatom *Seminavis robusta*): a cyclic heptapeptide featuring a β-sulfated aspartate^31^.

Our own research endeavours in this field started almost 40 years ago. Separation of a methanolic extract from the corpora cardiaca of the fruit beetle *Pachnoda sinuata* on reversed-phase high-performance liquid chromatography (RP-HPLC) resulted in two peaks with Trp fluorescence, with the larger polar peak sequenced as an octapeptide (code-named Melme-CC: pGlu-Leu-Asn-Tyr-Ser-Pro-Asp-Trp-NH_2_). The second and smaller peak gave clear signals in Edman degradation (after removing the N-terminal pGlu enzymatically) for all amino acids except position 6: (pGlu-Ile-Asn-Leu-Thr-Xaa-Gly-Trp)^32,33^. It was suspected but not proven that the amino acid at position 6 had undergone a modification. We had experienced the phenomenon of failure of detection of an amino acid during Edman degradation before, in the protea beetle *Trichostetha fascicularis*^18^. The peptide in question also gave no signal at position 6 but LC coupled to electrospray ionisation mass spectrometry (ESI-MS) detected a phosphorylated Thr residue (also called pT or pThr below) at that position. Thus, the protea beetle synthesised the unusual octapeptide, code-named Trifa-CC (pGlu-Ile-Asn-Met-Thr-pThr-Gly-Trp-NH_2_) in its CC.

At that time (1997) we never got a satisfactory mass spectrum from the *P. sinuata* peptide and, thus, could not deduce whether or not the PTM was Thr phosphorylation. Recently, we identified a peptide with the sequence proposed for the unresolved *P. sinuata* AKH but with an unmodified Thr at position 6 in the Eurasian bee beetle *Trichius fasciatus* and called it Pacsi-AKH (pGlu-Ile-Asn-Leu-Thr-Thr-Gly-Trp-NH_2_)^34^. Moreover, in another scarab beetle species, *Tropinota hirta*, a mass ion peak at *m/z* 994.431 was detected that “lost 80 mass units easily during MS analysis - even when no collision energy was applied”; the resulting peptide at *m/z* 914.468 had the same sequence as Pacsi-AKH^34^. This intriguing result, suggesting a phosphorylated or sulfated peptide, renewed our interest in analysing this and other extracts from cetoniid and dynastid beetles. After careful investigations, we clearly identify sulfated Thr-containing peptides - not proteins - for the first time in nature in 11 beetle species and describe here the validation of the sulfoThr^6^ sequences with manually synthesised sulfated peptides.

## Materials and methods

### Insects

Adult specimens of various insect species were used. They were of unknown age, and we did not distinguish between sexes. The scarab beetles (order Coleopteran family Scarabaeidae) of the subfamily Dynastinae were collected on an organic orange farm, Rosedale, close to Addo, South Africa (*Oryctes boas*), or in a private garden in Mowbray/Cape Town, South Africa (*Syrichthodontus spurius)* or in a vineyard of the Pinzenet H. Hummel in Villany, Hungary (*Pentodon idiota*). *Xylotrupes gideon* was purchased from a commercial breeder. Scarabs of the subfamily Cetoniinae were collected in a private garden in Mowbray/Cape Town, South Africa (*Pachnoda sinuata* and *Dicronorhina derbyana derbyana*) and on the paddocks and wooden area of a horse riding school in Poštorná, Czech Republic (*Tropinota hirta*,* Trichius fasciatus*,* Protaetia cuprea*,* Cetonia aurata* and *Oxytherea funesta).* The heteropteran true bug (order Hemiptera) of the family Coreidae, *Holopterna alata*, came from a private garden in Mowbray/Cape Town, South Africa. None of the species are on the list of the most-recent International Union for Conservation of Nature’s Red List of the critically endangered, threatened or protected species (https://www.iucnredlist.org/; accessed 22.02.26).

### Tissue preparation and peptide isolation

The CC glands from the various insect species were dissected directly after collection or arrival from the supplier with the aid of a stereomicroscope at 20 to 40-fold magnification. Glands from the same species were pooled in a microcentrifuge tube containing 400 µL of 80% v/v methanol and were extracted by approved methods and dried in a vacuum centrifuge^35^.

### Peptide separation and sequence characterisation by LC-MS/MS

Peptides were analysed with nano RP-LC coupled to high-resolution MS as described previously^36^ using Synapt G2 Si and M-Class instruments (Waters Corp., Manchester, UK). Briefly, AKH candidate peaks were identified by target-MS/MS on the singly-charged peptide ion in positive ion mode and data were analysed manually. Accurate mass measurement was performed to distinguish modifications close in mass. Peptides were validated using their synthetic counterparts in the same manner. Retention time (RT) and fragment ion spectra had to match for successful identification. Selected examples are provided (see Figures in the Results section). Spectra labelling follows the nomenclature used by the Waters MassLynx software; the fragment ion tables for the spectra are available in the Supplementary Information section for further explanation.

### Synthetic peptides

1. Phosphorylated peptide: The phosphorylated form of the peptide Schgr-AKH-II (i.e. pGlu-Leu-Asn-Phe-Ser-pThr-Gly-Trp-NH_2_), was synthesised by Dr K.D. Clark (Department of Entomology, University of Georgia, Athens, USA) according to the same procedure as outlined in detail for the beetle peptide Trifa-CC^18^.
2. Sulfated peptides: In total, 6 peptides were synthesised (3 standard AKH octapeptides and their corresponding Thr^6^ sulfated analogues) manually in fritted polypropylene syringes using Fluoren-9-ylmethoxycarbonyl (Fmoc)-strategy solid-phase peptide synthesis (SPPS) on either 25 or 50 µmol scale. Fmoc-protected l-amino acids, coupling reagents and resins (Rink amide AM, 100–200 mesh) were purchased from Mimotopes and Novabiochem. A sulfated Fmoc-protected l-Thr amino acid was synthesised with a tetrabutyl ammonium counter-ion (Fmoc-Thr(SO_3_^−^N^+^Bu_4_)-OH) using a procedure described by^37^. It is important to note that the tetrabutyl ammonium salts offer acid stability to the otherwise highly acid-labile sulfates, providing access to peptides bearing sulfated Thr residues by Fmoc SPPS protocols.

Typically, Fmoc SPPS involves three steps performed iteratively to assemble the full-length peptides. These steps include:

*(i) Fmoc deprotection*: The resin-bound peptide was shaken in a solution of 20 vol% piperidine in dimethylformamide (DMF, 2 mL, 5 min). The deprotection solution was then drained and the process was repeated one more time. The deprotection solution was drained again and the resin was washed with DMF (4 × 4 mL). (*ii) Coupling*: The resin-bound peptide was shaken in a solution of Fmoc-AA-OH (4 equivalents (eq.)., 0.2 M), Oxyma [ethyl 2-cyano-2-(hydroxyimino)acetate] (4.4 eq., 0.22 M) and DIC (*N*,*N*′-diisopropylcarbodiimide) (4 eq., 0.2 M) in DMF for 45 min at room temperature. The coupling solution was drained, and the resin was retreated with a fresh coupling solution and finally washed with DMF (4 × 4 mL). *iii) Capping*: The resin-bound peptide was shaken in a solution of 5 vol% acetic anhydride (Ac_2_O) and 10 vol% DIPEA (*N*,*N*-diisopropylethylamine) in DMF (2 mL, 5 min). The capping solution was then drained, and the resin was washed with DMF (4 × 4 mL).

Acidolytic cleavage of peptides from resin with deprotection of side-chain protecting groups: After complete elongation of the desired peptide, the resin was washed with CH_2_Cl_2_ (2 × 3 mL), and treated with a mixture of trifluoroacetic acid (TFA), tri*iso*propylsilane (TIS) and water (18:1:1 v/v/v, 2 mL) was added to the resin-bound peptide (25 µmol) and agitated at room temperature for 2 h. For the sulfated peptides, the cleavage time was shortened to 30 min to minimise premature loss of the sulfate ester moiety. The resin was then filtered, and the cleavage cocktail was concentrated under nitrogen flow. Diethyl ether (40 ml) was added and the suspension cooled to 0 °C for 10 min. The precipitate was pelleted by centrifugation at 6800 rcf for 10 min at 0 °C and the supernatant decanted. The crude peptide was dissolved in DMSO and purified using RP high-performance LC with a Waters 600 multisolvent delivery system and Waters 500 pump with 2996 photodiode array detector or Waters 490E Programmable Wavelength Detector operating at 214 and 280 nm on a preparative C18 column (Waters X-Bridge^®^ C18, 130 Å, 19 × 150 mm column, with a flow rate of 10 or 14 mL min^-1^). For the unmodified peptides, 0.1 vol% TFA in water (solvent A) and 0.1 vol% TFA in MeCN (solvent B) was used as mobile phase on linear gradients unless otherwise specified, while 0.1 vol% NH_4_OH was used as an additive for the sulfated peptides.

The purity and authenticity of the final synthetic peptides was confirmed using analytical LC and LC-MS, respectively. Analytical LC traces were obtained using a Waters Acquity UPLC system equipped with a PDA el detector (l = 210–400 nm), a sample manager FAN and Quaternary Solvent Manager (H-Class) modules. Separations were performed using a C18 column (Waters Acquity BEH, 130Å, 1.7 μm, 2.1 mm × 50 mm) operating at 0.6 mL min^-1^ with 0.1 vol% TFA as the mobile-phase additive for unmodified peptides and 0.1 vol% formic acid for the sulfated peptides. LC-MS analysis was performed on a Shimadzu 2020 UPLC-MS instrument using a Nexera X2 LC-30AD pump, Nexera X2 SPD-M30A UV/Vis diode array detector, and a Shimadzu 2020 (ESI) mass spectrometer operating in positive and negative mode. Sulfated peptides were analysed using negative ESI-MS. *Note*: Please refer to the supporting information file for yields and characterisation details of all the synthetic peptides (Supplementary Figures S1–S6).

### Retrieving AKH sequences from publicly available data bases

The primary sequence of AKH family peptides from leaf-footed bugs (Hemiptera), flower chafers (Coleoptera, subfamily Cetoniinae) and rhinoceros beetles (Coleoptera, subfamily Dynastinae) were “mined” from the National Center for Biotechnology Information datasets (https://blast.ncbi.nlm.nih.gov/) via homology searches using BLAST (Basic Local Alignment Search Tool). Potential AKH translated sequences obtained in this way from genomes, transcriptomes and bioprojects are indicated in the current study with the accession or sequence identity number from NCBI supplied in parenthesis.

## Results

### Pacsi-AKH

**Fig. 1:**
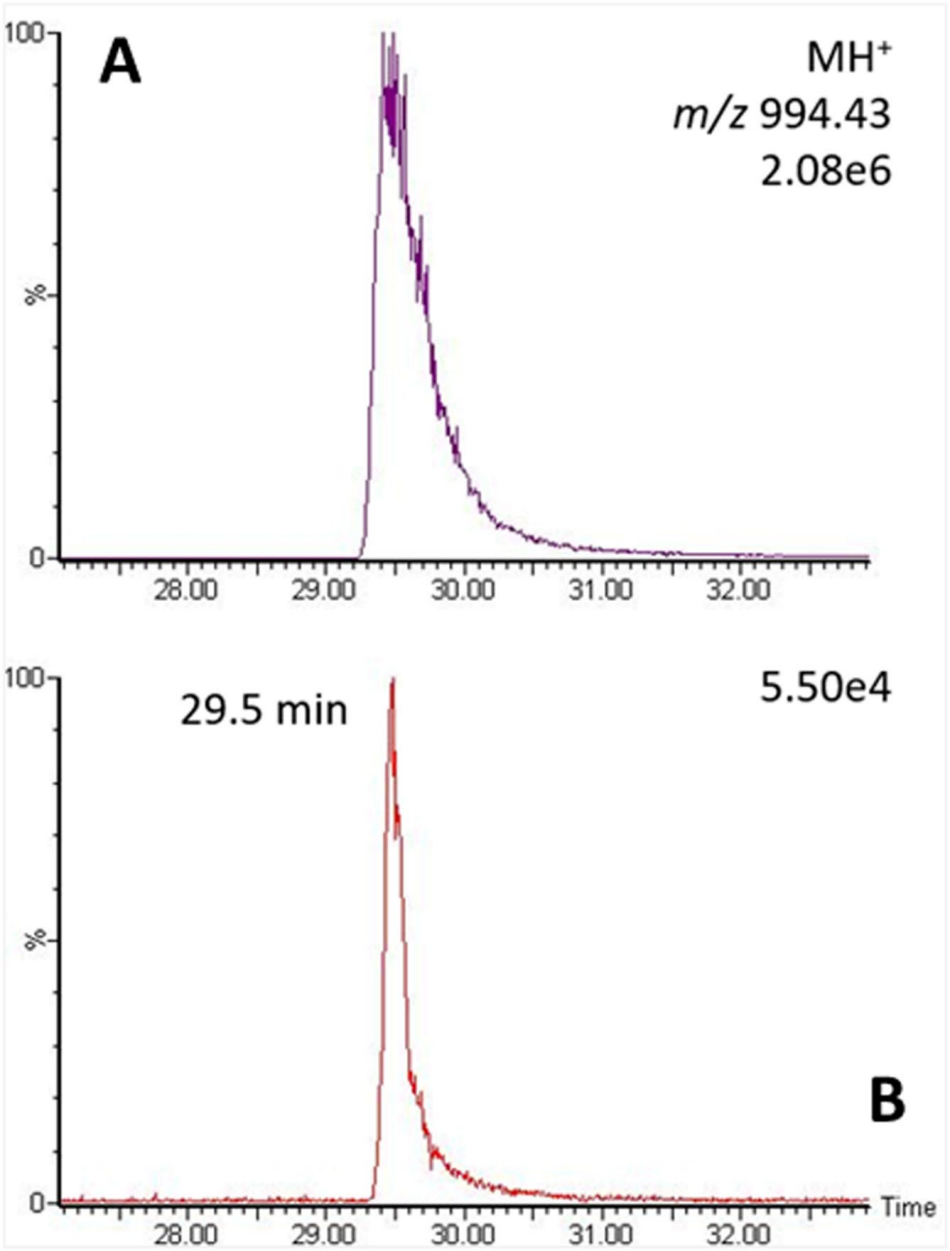
LC-traces of the (**A**) synthetic peptide, sulfated Pacsi-AKH with the sequence pGlu-Ile-Asn-Leu-Thr-sThr-Gly-Trp-NH_2_, and (**B**) native sulfated Pacsi-AKH detected in CC extract from *T. hirta.* For MS spectrum of the endogenous peptide, see Fig. 6 in^34^.

The combined previous data on three species of cetoniid beetles (*P. sinuata*, *T. hirta* and *T. fasciatus*) prompted us to examine these species in more detail and, most importantly, to synthesise the peptide with a sulfate moiety at the Thr^6^ position where Edman degradation and MS/MS had previously not provided evidence for the PTM^33,34^. The synthetic sulfated Pacsi-AKH had the same retention time (RT) as the native peptide detected in the *T. hirta* CC extract (Fig. 1) with a strong signal at *m/z* 994.431, that lost 80 mass units for both the synthetic and the endogenous peptide during the MS experiment (for spectrum of the endogenous peptide, see Fig. 6 in^34^. It exhibited sodiated and potassiated satellite peaks commonly observed for AKHs^38^. The product peak at *m/z* 914.468 was accompanied by a loss of the elements of ammonia rather than water indicating the presence of a C-terminally amidated peptide^36^.

**Fig. 2:**
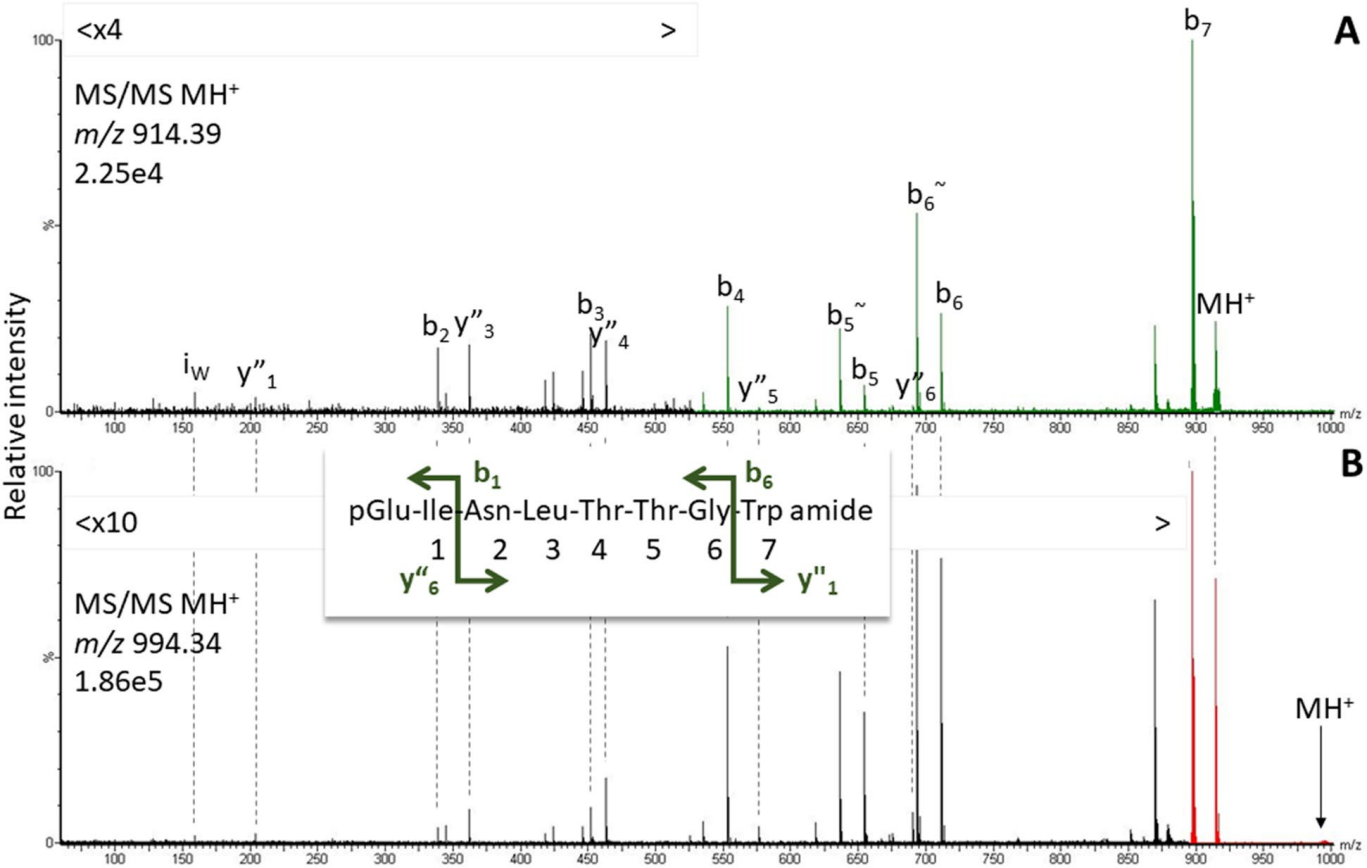
CID spectra of a pair of related peptides assigned to sequence pGlu-Ile-Asn-Leu-Thr-Thr-Gly-Trp-NH_2_ detected in the CC extract of *T. hirta.* (A) unmodified form (*m/z* 914.39) and (**B**) modified form (*m/z* 994.34). A peptide with the same derived primary sequence from CC of *Trichius fasciatus* is code-named Pacsi-AKH^34^.

**Fig. 3:**
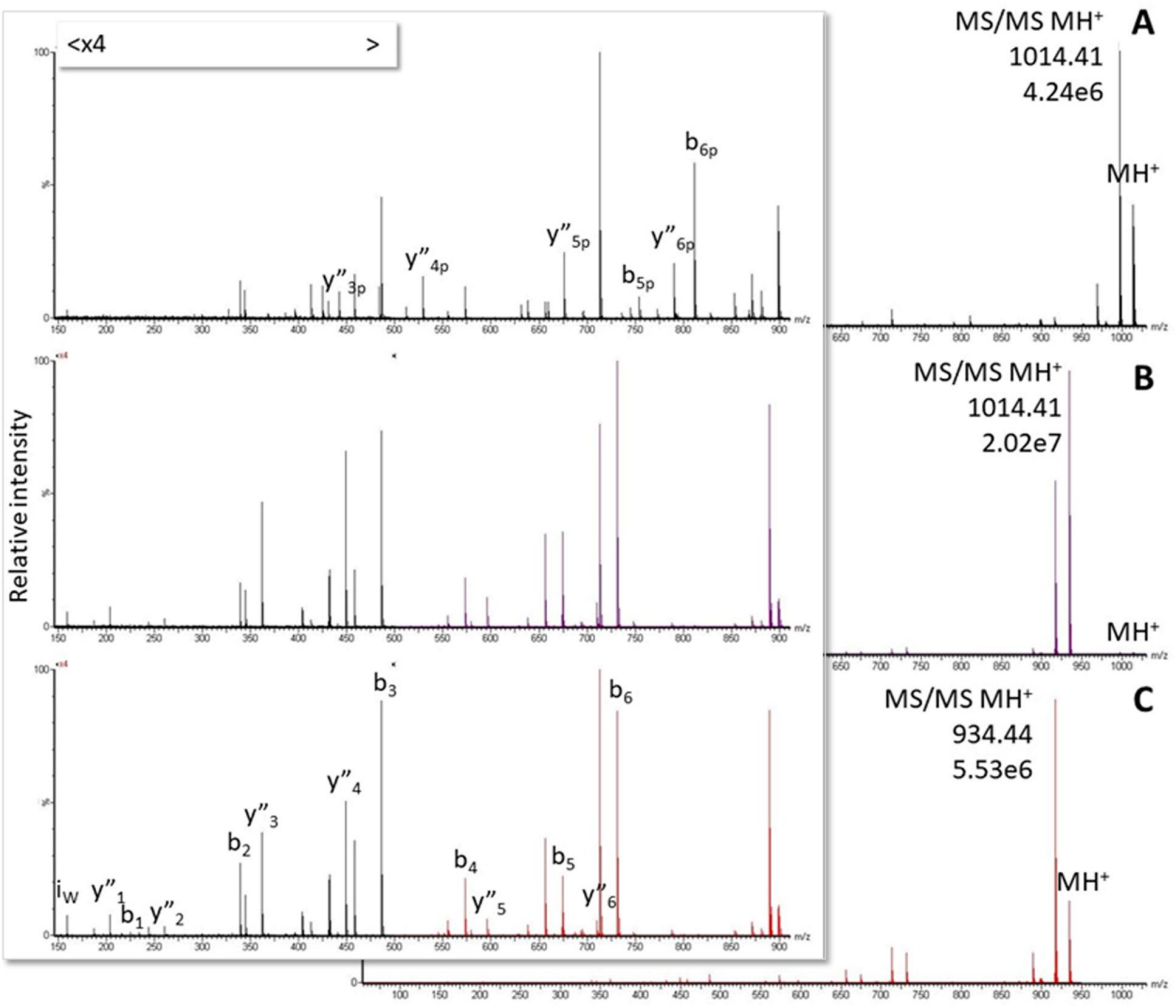
CID spectra of variously modified synthetic Schgr-AKH-II (pGlu-Leu-Asn-Phe-Ser-Thr-Gly-Trp-NH_2_) peptides: (**A**) phosphoThr^6^, (**B**) sulfoThr^6^, and (**C**) unmodified. The inset is a zoom into the range below *m/z* 900 for improved visualisation. The fragment ions for the sulfated peptides essentially match those of the unmodified peptide following the ready loss of SO_3_, while for the phosphorylated peptide, ions still carrying the phosphate moiety can be observed (label “p”). For theoretically expected ions, see Supplementary Fig. S7.

The modified peptide in *T. hirta* CC extract dominated in intensity over the unmodified form, which could hardly be distinguished from the ion of the same *m/z* value generated by ion source decay (neutral loss) in the instrument; it eluted (in only minimal quantities) less than a minute later than the sulfated species in LC. Gas phase fragmentation of the peaks for the modified and unmodified peptide form confirmed the assignment to the sequence for Pacsi-AKH (Fig. 2), i.e. both ion peaks exhibited the fragment ions expected for the unmodified peptide; fragment ions containing a sulfate moiety were not observed from the sulfated Pacsi-AKH. The 80 Da mass difference between the pair of related peptides shown in Fig. 2 hinted at either phosphorylation or sulfation, and we determined the latter by exact mass measurement (theoretical MH^+^ for sulfated peptide 994.4304, phosphorylated peptide 994.4399). Sulfated peptides have been shown before to undergo gas phase rearrangement with elimination of SO_3_ during collision-induced dissociation (CID) resulting in essentially identical fragmentation patterns compared to the unmodified species, which allows peptide sequence assignment but not the determination of the modified amino acid residue^30,39,40^. We confirmed this effect when measuring a synthetic AKH peptide, which we had available in phosphorylated and sulfated form (Fig. 3). While for the Thr^6^-phosphorylated Schgr-AKH-II, the important six ions of the b- and y-ion series, which were necessary for assignment of the modified site, were detected (Fig. 3A), this was not the case for the Thr^6^-sulfated species (Fig. 3B; Supplementary Fig. S7).

The analyses of additional CC extracts from cetoniid beetles (*D. derbyana derbyana*,* P. sinuata*,* P. cuprea*,* C. aurata*,* O. funesta*) resulted in the detection of a signal for modified Pacsi-AKH (Table 1), but there was no indication of significant quantities of the unmodified form. As mentioned for *T. hirta* above, only a very small signal for the unmodified peptide was detected or none at all, which can be the result of low concentrations below the detection limit (see data for Penid-AKH and Schgr-II-AKH below).

**Table 1:** Species investigated in the present study and the detected AKHs (MW – molecular weight; sulf. – sulfated; hyp. – proline hydroxylation).

Taxonomy	Subfamily	Species	AKH	Sequence *	MW
Coleoptera Polyphaga Scarabaeoidea Scarabaeidae	Cetoniinae	*Tropinota hirta* ^34^	Pacsi-AKH	pQINLTTGW amide	913.4658
			Pacsi-AKH sulf.	pQINLTsTGW amide	993.4226
			Melme-CC	pQLNYSPDW amide	1003.4400
			Melme-CC hyp.	pQLNYSHypDW amide	1019.4349
		*Pachnoda sinuata*	Pacsi-AKH sulf.	pQINLTsTGW amide	993.4226
			Melme-CC	pQLNYSPDW amide	1003.4400
		*Dicronorhina derbyana derbyana*	Pacsi-AKH sulf.	pQINLTsTGW amide	993.4226
			Melme-CC	pQLNYSPDW amide	1003.4400
		*Trichius fasciatus*	Pacsi-AKH	pQINLTTGW amide	913.4658
			Melme-CC	pQLNYSPDW amide	1003.4400
		*Protaetia cuprea*	Pacsi-AKH sulf.	pQINLTsTGW amide	993.4226
			Melme-CC	pQLNYSPDW amide	1003.4400
		*Cetonia aurata*	Pacsi-AKH sulf.	pQINLTsTGW amide	993.4226
			Melme-CC	pQLNYSPDW amide	1003.4400
		*Oxythyrea funesta* ^34^	Pacsi-AKH sulf.	pQINLTsTGW amide	993.4226
			Melme-CC	pQLNYSPDW amide	1003.4400
			Melme-CC hyp.	pQLNYSHypDW amide	1019.4349
	Dynastinae	*Pentodon idiota*	Penid-AKH	pQVNISTGW amide	885.4345
			Penid-AKH sulf.	pQVNISsTGW amide	965.3913
			Dorpa-AKH	pQVNYSPVW amide	973.4658
		*Xylotropes gideon*	Penid-AKH	pQVNISTGW amide	885.4345
			Penid-AKH sulf.	pQVNISsTGW amide	965.3913
		*Oryctes boas*	Penid-AKH	pQVNISTGW amide	885.4345
			Penid-AKH sulf.	pQVNISsTGW amide	965.3913
		*Syrichthodontus spurius*	Penid-AKH	pQVNISTGW amide	885.4345
			Penid-AKH sulf.	pQVNISsTGW amide	965.3913
Hemiptera, Heteroptera, Coreidae	Coreinae	*Holopterna alata*	Schgr-AKH-II	pQLNFSTGW amide	933.4345
			Schgr-AKH-II sulf.	pQLNFSsTGW amide	1013.3913

*pQ – pyroglutamate; sT – sulfoThr; Hyp - hydroxyproline.

Sulfation and the exact site of sulfation could not be determined directly from MS/MS sequencing in any of the beetle species investigated as none of the resulting fragments carried the sulfate moiety. However, proof of the sulfated residue is supplied indirectly by observing the exact same sequencing behaviour and RT of the native modified Pacsi-AKH and a synthetic sulfated Pacsi-AKH peptide as shown, exemplary, for *D. derbyana derbyana* (Supplementary Fig. S8; Supplementary Fig. S9 for the expected fragment ions of Pacsi-AKH).

### Schgr-AKH-II

**Fig. 4:**
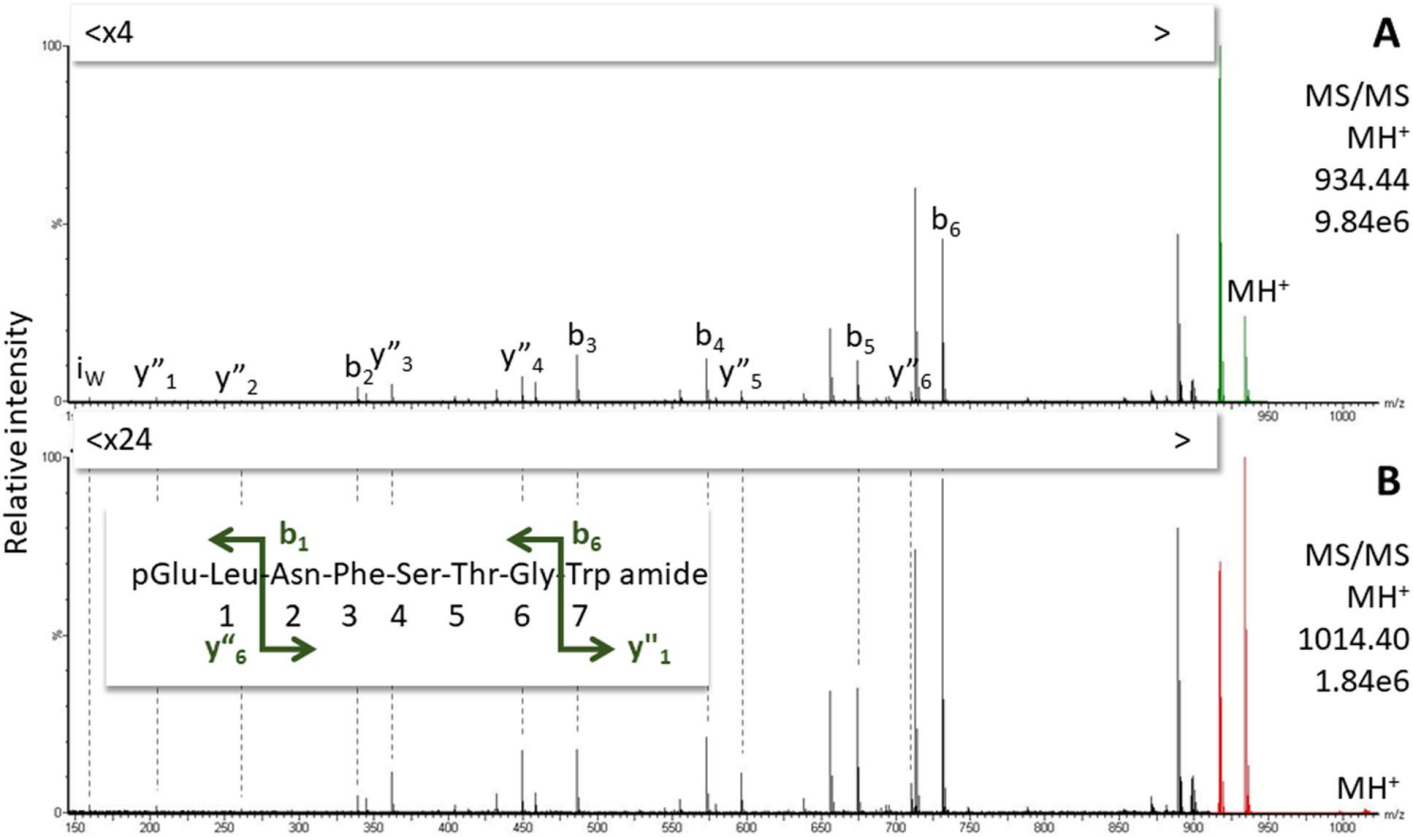
CID spectra of the unmodified (**A**) and sulfated (**B**) Schgr-AKH-II detected in the CC extract of *H. alata*. Compare with Fig. 3 for spectra of the respective synthetic peptides. The fragment ions of both native *H. alata* peptides in the CC match to those of the synthetic peptide counterparts. For theoretically expected ions, see Supplementary Fig. S7.

Studying the AKHs of the coreid twig wilter *H. alata*, we also encountered ion species differing by 80 mass units, which could be assigned to a modified form of the well-known peptide Schgr-AKH-II. Schgr-AKH-II was first identified by different methods in locusts of the genus *Schistocerca*^41,42^ and had been shown to occur in *H. alata*^43^. The validation experiment with synthetic sulfated Schgr-AKH-II in the current study showed the exact tandem mass spectra (Figs. 3B and 4) as for Schgr-AKH-II. Moreover, we demonstrated that the fragmentation behaviour of the phosphorylated AKH was significantly different from that of the sulfated species (Fig. 3): CID of phosphorylated Schgr-AKH-II (Fig. 3A) gave major b and y ions that still had the phosphate moiety attached and thus indicated the Thr^6^ residue as modified, whereas sulfated Schgr-AKH-II did not exhibit modified fragment ions (Figs. 3B and 4B). Thus, the mass of + 80 Da and the CID profile of a peptide can be a reliable discriminator between the type of PTM in AKHs, i.e. between phosphorylation and sulfation. Both the modified and the unmodified peptide were present in the CC extract with the former eluting 1.4 min before the latter in LC due to increased polarity.

### Penid-AKH

**Fig. 5:**
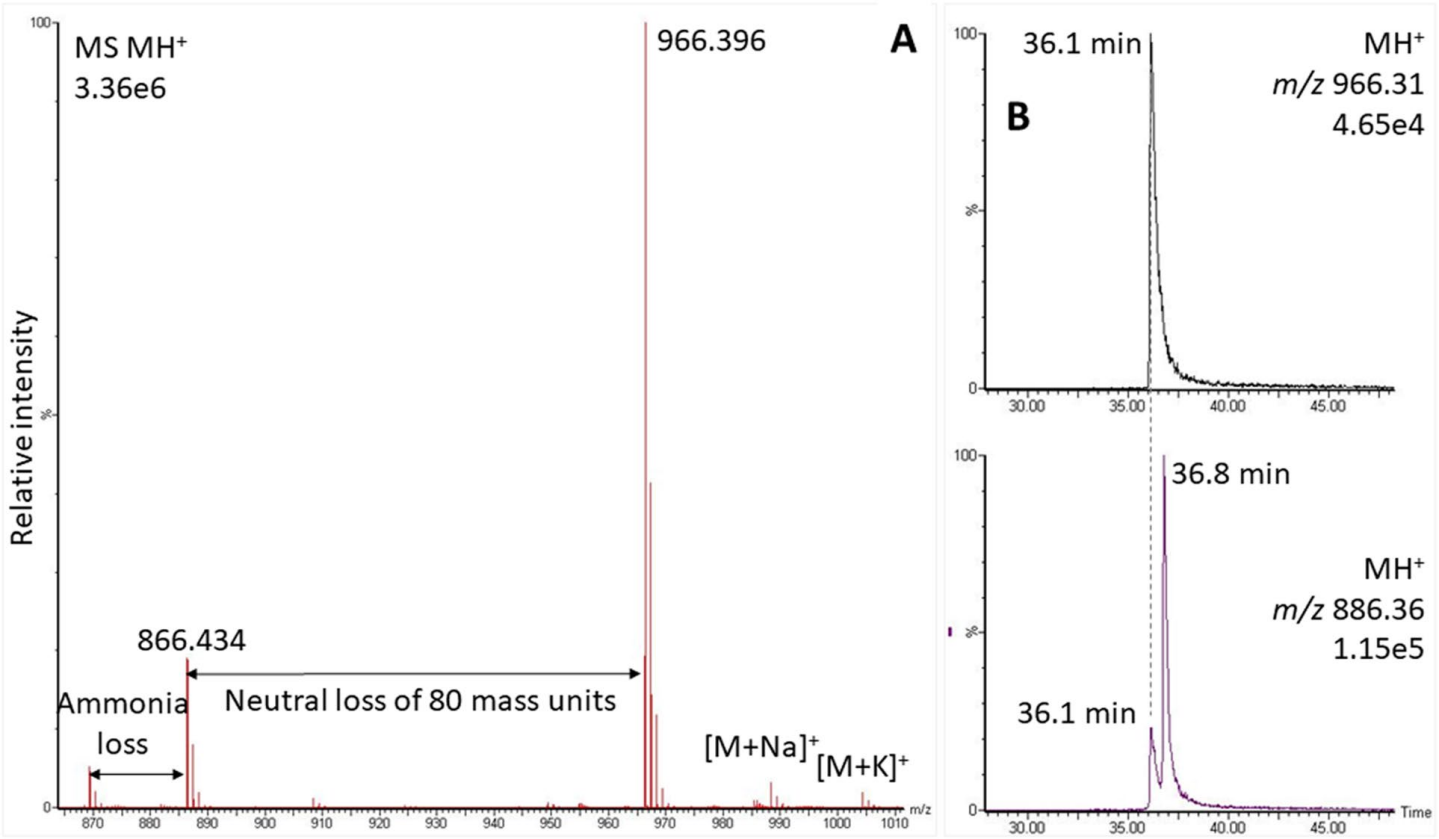
MS spectrum (**A**) and LC-MS trace (**B**) for a modified and unmodified peptide pair, validated as sulfated Penid-AKH and Penid-AKH, respectively, detected in *P. idiota* CC extract. Shown is the peak for the modified ion species (sulfated), that easily lost 80 mass units, with satellite ions and neutral losses, eluting shortly before the unmodified species. Penid-AKH is a novel AKH with the sequence of pGlu-Val-Asn-Ile-Ser-Thr-Gly-Trp-NH_2_.

**Fig. 6:**
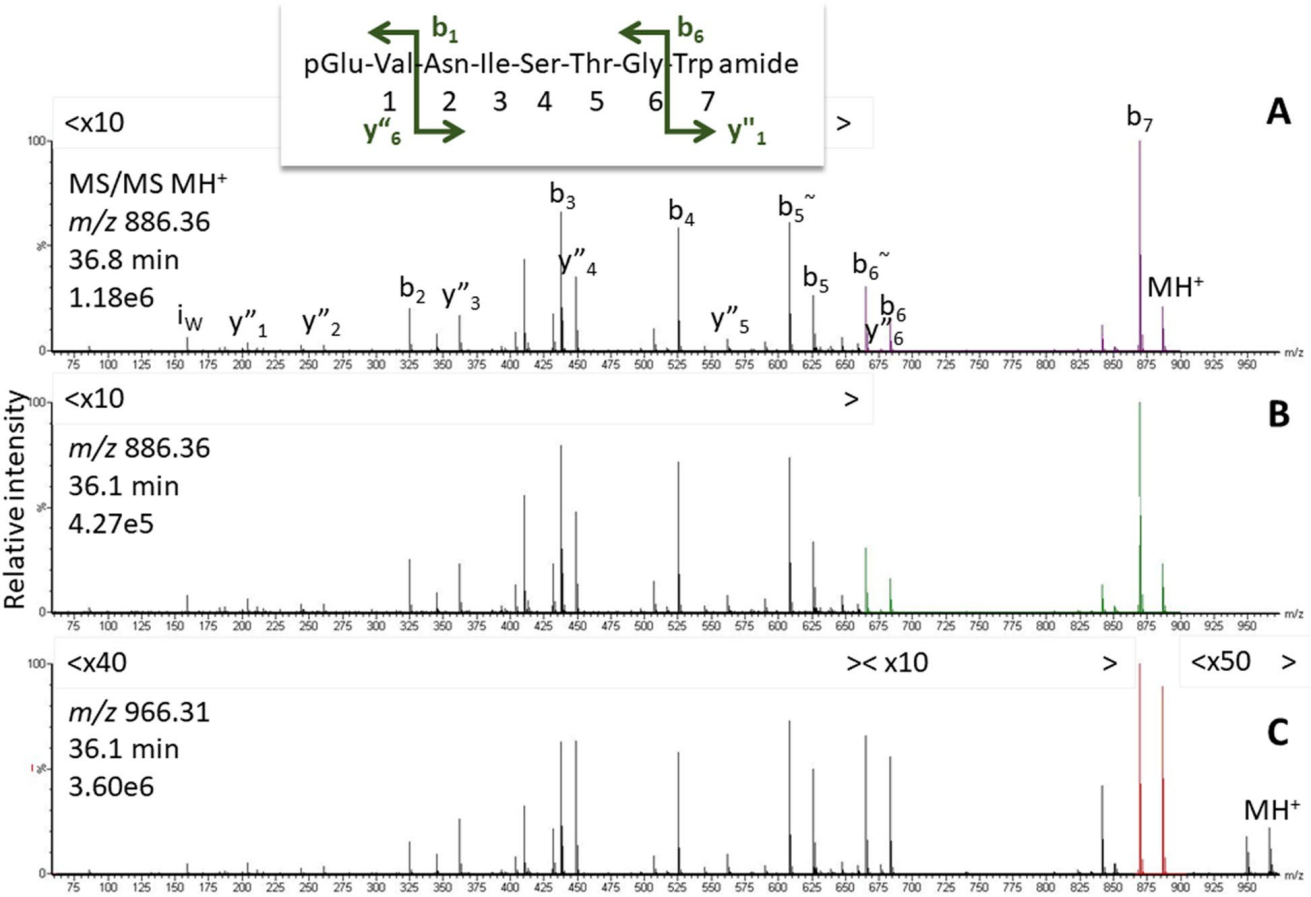
CID spectra of peaks detected in *P. idiota* CC extract for a peptide pair assigned to primary sequence pGlu-Val-Asn-Ile/Leu-Ser-Thr-Gly-Trp-NH_2_ and validated as Penid-AKH (Ile^4^) and sulfated Penid-AKH (Ile^4^, sulfoThr^6^). (**A**) Penid-AKH (unmodified species); (**B**) product ion profile of the smaller peptide species that originated by in-source decay from sulfated Penid-AKH; (**C**) “non-degraded” sulfated Penid-AKH. All peptide species (**A**–**C**) generated the same fragment ions that coincided with the mature peptide sequence for Penid-AKH; for calculated theoretically expected ions, see Supplementary Fig. S10.

Another group of beetles that we studied, scarabs of the subfamily Dynastinae, also presented with a modified AKH. We investigated four species in the current study and give exemplarily here the detailed data for *P. idiota*. In LC-MS analysis of the CC extract from *P. idiota*, we noted a signal at *m/z* 886.434, as well as a strong signal at *m/z* 966.396, which lost 80 mass units to a product peak at *m/z* 886.434 and exhibited a similar general mass profile (Fig. 5A) as discussed above for other AKH peptides. The larger peptide eluted slightly before the smaller species in LC (36.1 vs. 36.8 min, Fig. 5B). Gas phase fragmentation of the peaks detected at the different RT, as well as the *m/z* 886.434 product peak resulting from in-source decay of the peak measured at the same RT as the signal at *m/z* 966.396 confirmed that they were, indeed, related to each other (Fig. 6, Supplementary Fig. S10). As with the other sulfated AKHs presented above, under both low-energy conditions in the ion source of the mass spectrometer and CID provoked in the collision cell of the instrument, the selected ion peaks exhibited common fragment ions, which were tentatively assigned to the peptide sequence pGlu-Val-Asn-Ile/Leu-Ser-Thr-Gly-Trp-NH_2_. We determined sulfation by exact mass measurement (theoretical MH^+^ for sulfated peptide 966.3991, phosphorylated peptide 966.4068). To clarify the isobaric amino acid in position 4, we synthesised a peptide with Leu at position 4 and compared its RT with that of the native Penid-AKH on LC-MS; the RT did not match by about 0.5 min. A synthetic peptide with Ile at position 4 matched the RT of the native Penid-AKH at *m/z* 886.434; similarly, a synthetic sulfated peptide with Ile at position 4 matched the RT of the native AKH at *m/z* 966.31. The correct sequence for sulfated Penid-AKH is thus proven to be pGlu-Val-Asn-Ile-Ser-sThr-Gly-Trp-NH_2_.

Additionally, a peptide with 3 amino acid substitutions relative to Penid-AKH, was identified and sequenced from the CC extract of *P. idiota* and validated with a synthetic peptide to be the already-known Dorpa-AKH (Table 1). Dorpa-AKH (pGlu-Val-Asn-Tyr-Ser-Pro-Val-Trp-NH_2_) was not detected in CCs from other Dynastinae beetles in the current study, whereas peptide pairs corresponding to Penid-AKH and sulfated Penid-AKHs were detected in *Xylotrupus gideon*, *Oryctes boas* and *Syrichthodontus spurius* (Table 1). Penid-AKH and sulfated Penid-AKH were validated in these beetle species with the corresponding synthetic peptides as shown for *X. gideon* (Supplementary Fig. S11) and *S. spurius* (Supplementary Fig. S12).

## Discussion

Although there is a vast amount of literature available of the sulfation of Tyr in proteins and peptides^25,26^, the information on sulfation of the hydroxylated amino acids Thr and Ser is rarely described and only for larger proteins^30^. The current investigation is the first detailed report on short peptide hormones with a sulfoThr residue. It appears that the insect AKH family is prone to PTMs since proline hydroxylation, proline isomerisation, Trp-C mannosylation and phosphorylation have also been shown to occur to these peptide hormones (see Introduction). Is there something out of the ordinary in these AKH molecules? We do not think so. Our theory is that such PTMs do occur very likely in other bioactive peptides and peptide hormone systems but have not yet been detected due to those systems being under-investigated, whereas the AKH system has been examined in many different insect species over the past 50 years^6,8^. Additionally, genomic and transcriptomic information is not helpful in this respect and typically does not give information of the presence of such PTMs. These unusual PTMs came to light in our laboratory as a result of our ongoing research on AKHs, their activity and structure over several decades and with more than 100 bioforms (peptide structures elucidated from biological samples) now known.

When we discovered first the loss of 80 mass units during the structure elucidation work of some cetoniid beetles, we were inclined to believe that we had found a second case of phosphorylation of an AKH as previously for the cetoniid *T. fascicularis*^18^. Accurate mass measurement, however, indicated sulfation, which was confirmed in the current study by the peculiar CID behaviour of sulfated AKHs not showing any modified fragment ions. This is known from work on sulfoTyr, as well as sulfoThr and sulfoSer in proteins^30,39,40,44–46^. The remaining major question was which amino acid of the molecule was sulfated in the *P. sinuata* peptide since it contains two possible amino acids for sulfation: Thr at position 5 and 6. Earlier work in the late-1990s using Edman degradation sequencing had given a clear result for Thr at position 5 but no signal for the residue in position 6, suggesting that Thr^6^ was the modified residue^33^. A synthetically produced site-specifically sulfated peptide generated in the current work was, therefore, paramount to support the assignment of sulfoThr^6^ as having the same RT and CID pattern as the native peptide; this sulfated peptide of *P. sinuata* is referred to as sulfated Pacsi-AKH. Two further sulfoThr^6^-containing peptides are described from other insect species and similarly validated with synthetic sulfopeptide standards in the current investigation. Sulfated Penid-AKH occurs in the CC of a variety of Dynastinae beetles (current study) and sulfated Schgr-AKH-II that is found in the twig wilter, *H. alata* (current study &^19^). It should be noted that Penid-AKH and Schgr-AKH-II also have two amino acid candidates for sulfation, viz. Ser at position 5 and Thr at position 6, while MS clearly indicated that only one of these residues is sulfated. Although neither of these native sulfated peptides were subjected to Edman degradation in the current study, it is presumed that a “gap” in the sequence would have resulted as in the case of sulfated Pacsi-AKH^33^ and the labile nature of the O-sulfation^46^. Thus, based on analogy, the sixth amino acid (Thr) was selected here for modification when we prepared a synthetic standard of the sulfated Penid-AKH and sulfated Schgr-AKH-II: PTMs discovered on the sixth residue in other AKHs are sulfated Pacsi-AKH (current work), proline hydroxylation (^1,17^, phosphorylation^18^, and a possible *cis*–*trans* isomerisation^14^.

**Fig. 7:**
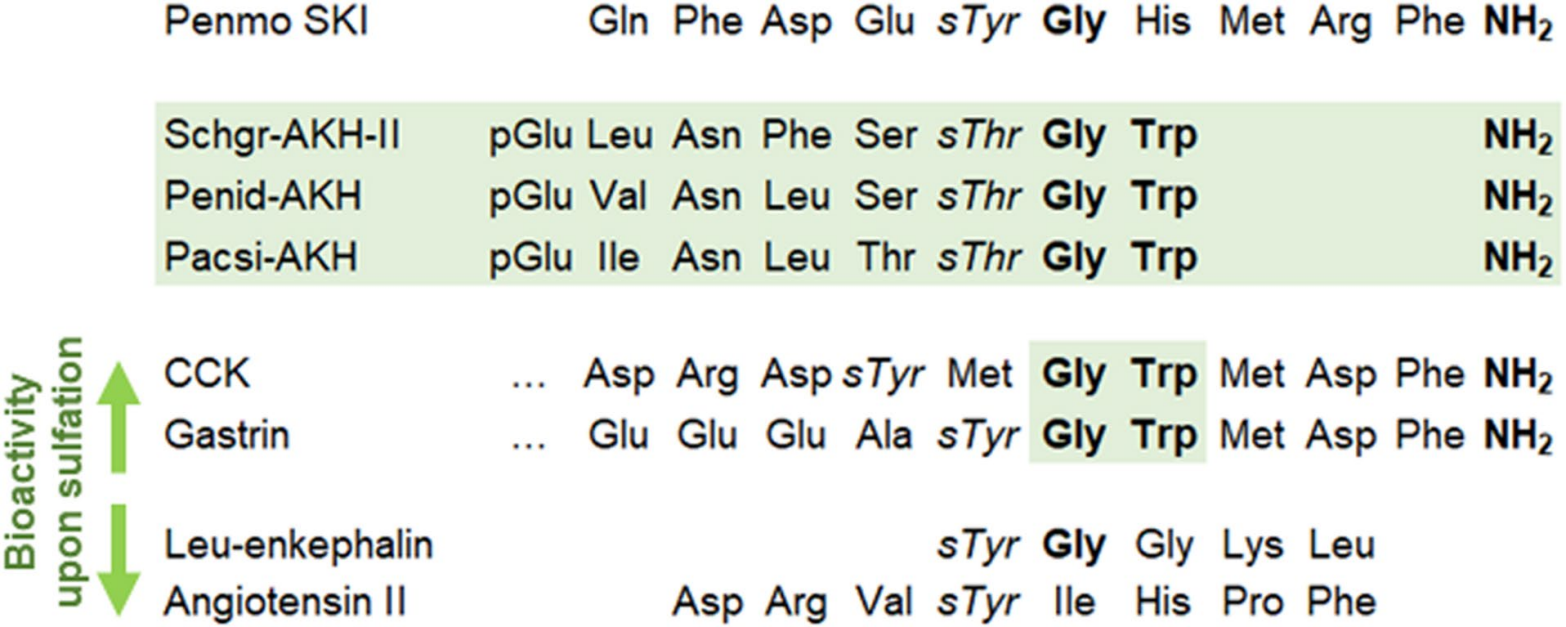
Comparison of terminal sequences of sulfoTyr (sTyr) neuroendocrine peptides CCK, gastrin, Leu-enkephalin and angiotensin II^65^ and sulfakinins from *Penaeus monodon*^46^ with the sulfoThr (sThr) AKHs of the current study. Shared features are printed in bold, modified sites in italic.

It is interesting to note that some features of the insect sulfoThr^6^ AKH peptides are reminiscent of some of the well-known sulfoTyr peptides of vertebrates (i.e. gastrin and CCK), such as the Gly-Trp sequence that follows the sulfated residue and the C-terminal amidation (Fig. 7), while in the arthropod sulfakinin peptides, the sulfoTyr residue is followed by Gly-His^46^. One might think that this is a characteristic for sulfation of Tyr and Thr, however, for Tyr sulfation the acidic amino acids such as Glu or Asp are established as the key motif for enzymatic transfer by tyrosylprotein sulfotransferases (TPSTs)^47,48^); thus, the observation could be coincidental. The TPST family of enzymes is located in the *trans*-Golgi network and whether the same TPST is responsible for sulfation of Ser and Thr is not known^30,49^ and remains to be shown. Sequence homology between TPSTs from numerous animal species, including vertebrates, arthropods (crustaceans and insects) and other invertebrates, are sufficiently high that searches of EST databases and genomes reveal TPST orthologue sequences^49–51^.

In CCK and gastrin, Tyr sulfation is essential for full biological activity^52^, while in Leu-enkephalin and angiotensin II, bioactivity is significantly reduced by sulfation^53,54^. In insects the sulfated tyrosine in sulfakinins is also largely important for physiological function as shown in biological assays for inhibition of food intake and for myotropic action, although the non-sulfated peptide equivalent may have partial activity^55^. The importance of Tyr sulfation has also been demonstrated in receptor activation studies where the sulfakinin peptide was 3000-fold more potent in activating the sulfakinin receptor of *Drosophila melanogaster* compared with non-sulfated sulfakinin analogues^55^. Similar results were obtained with the sulfakinin signalling system of the oyster *Crassostrea gigas*^56^. Determining the functional consequence of the sulfoThr modification of the AKHs versus unmodified AKH templates will therefore be an important avenue of future research efforts.

Apart from the hitherto unusual sulfoThr PTM, two of the AKHs in our current study that serve as precursors for sulfation, viz. Pacsi-AKH and Penid-AKH, present another peculiarity, i.e. the absence of an aromatic amino acid in position 4 of the peptide chain. Instead of the hallmark Phe or the rarer Tyr at position 4^34^, a Leu residue (Pacsi-AKH) or an Ile residue (Penid-AKH) is present (Table 1). In the phosphorylated Trifa-CC peptide of another cetoniid beetle, Met replaces Phe in position 4^18^ and Met^4^ is also found in the peptide code-named Ampso-AKH-II which is synthesised in the CC of the scarab beetle *Amphimallon solstitiale* (Subfamily: Melolonthinae)^34^. This begs the question whether these peptides are “true” AKH members? To answer this question comprehensively one would have to find a specific receptor that would only bind Pacsi-AKH (or Penid-AKH) but not any of the conventional Phe-containing AKHs. This may be done via in vitro receptor assays or via in vivo metabolic assays together with a very similar “classic” AKH to Pacsi-AKH and Trifa-CC, such as Euoin-AKH (pGlu-Ile-Asn-Phe-Thr-Thr-Gly-Trp-NH_2_) that was identified in the dung beetle species *Euoniticellus intermedius* (Family: Scarabaeidae^57^), or use Grybi-AKH (pGlu-Val-Asn-Phe-Ser-Thr-Gly-Trp-NH_2_) from the cricket *Gryllus bimaculatus* (Family: Gryllidae^58^) in place of Penid-AKH, or Schgr-AKH-II (pGlu-Leu-Asn-Phe-Ser-Thr-Gly-Trp-NH_2_) in place of Ampso-AKH-II. Currently, in vitro receptor assays are not possible since, to our knowledge, no cetoniid or dynastid beetle AKH receptor has been identified. From in vivo biological assays we know, however, that the phosphorylated Met^4^ containing Trifa-CC is biologically active in the protea beetle and in the fruit beetle *P. sinuata*, whereas the migratory locust (*L. migratoria*) and American cockroach (*P. americana*) did not react with substrate mobilisation upon injection of this peptide^18^. This result suggests that Trifa-CC is a true AKH peptide recognised by the AKH receptor of its own species but not by receptors of those insects that produce conventional (Phe^4^ containing) AKHs. To test the currently described peptides functionally is immensely difficult because the donor species are not held in culture, and collection in nature results in too few specimens for detailed and statistically relevant bioassays. The way forward would be to identify and clone the AKH receptor of one of the cetoniid beetle species and perform in vitro receptor activation assays with native and carefully designed synthetic peptides to find out which amino acids are essential for receptor recognition. Currently only a handful of beetle AKH receptors have been genomically characterised and studied in some detail^59–61^. Thr sulfated proteins in animals are thought to act as modulators of protein-protein interactions, such as in structural assembly and signalling^30^. A comprehensive study of the human CCK_A_ receptor is reported on^62^ where (amongst others), chemical modelling was conducted to reveal which receptor sites are involved in ligand recognition, binding and selectivity, i.e. discriminating between sulfated and non-sulfated CCK peptides. Such receptor-ligand models of the beetle AKHR will be useful to understand whether Thr sulfation is necessary for binding and activating the AKH receptor of insects.

From a taxonomic point of view it is interesting to note that the rhinoceros beetles (Family: Dynastinae) of the current study belong all to different tribes and yet produce the same peptide, Penid-AKH and its sulfated form (Table 1): *P. idiota* is a member of the largest tribe Pentodontini, *O. boas* belongs to the Oryctini, *X. gideon* to the Dynastini (the true rhinoceros beetles) and *S*. *spurius* to the Phileurini tribe^63^. *P. idiota*, additionally synthesises Dorpa-AKH (current study). While only Melme-CC is reported present in the CC of the rhinoceros beetle, *Oryctes rhinoceros*^64^, we suggest that Penid-AKH is co-produced, as gleaned from their MALDI-MS data (Fig. 4 in^64^]) that show two sets of sodiated and potassiated pairs corresponding to Melme-CC and possibly Penid-AKH. This occurrence of one unvariant AKH sequence structure in the scarab beetle subfamily Dynastinae plus one variant is reminiscent of the situation in the subfamily Scarabaeinae which in all but one tribe Scade-CC-I is synthesised as unvariant form next to a variant form (either Scade-CC-II, Cirba-AKH or Oniay-CC)^57^.

The bioinformatic searches that we performed with specific search criteria revealed a relatively restricted species dataset of the insects covered in the current study. Nevertheless, for the Coreidae bugs we find Schgr-AKH-II predicted from the genomes of *Homoeocerus unipunctatus* (Sequence ID: SRA: SRR34847808.16059882.2) and *Leptoglossus phyllopus* (Sequence ID: SRA: SRR28382981.8891177.1) which coincides with the sequence data of *H. alata*. Similarly, in the Cetoniinae genomes or transcriptomes of *Protaetia brevitarsis*,* Cetonia aurata* and *Osmoderma eremita*, a preprohormone sequence for Melme-CC is encoded (SRA: DRR672780, OX421884.1, and JARFOC010030109.1, respectively), which fits with the current findings (Table 1), although we have, thus far, not identified a preprohormone of Pacsi-AKH. For the Dynastinae, 5 species have Penid-AKH encoded, viz. *Oryctes rhinoceros* (JAIOKQ010000240.1), *Oryctes borbonicus* (LR736864.1), *Allomyrina (Trypoxylus) dichotomus* (CM042076.1), *Dynastes hercules hercules* (JBNAZS010000003.1), *Dynastes hercules reidi* (JBNAZT010000003.1), that again, complements the chemical sequencing data of the current study. Sulfation as a post-translational modification is not encoded in the nucleotide sequences.

## Supplementary Information

Below is the link to the electronic supplementary material.

Supplementary Material 1

## Data Availability

All data generated or analysed during this study are included in this published article (and its Supplementary Information files).
