# Supplementary material for: Threonine sulfation: a rare post translational modification in insect adipokinetic hormones
Source: Sci Rep. 2026 May 15;16:22190. doi: 10.1038/s41598-026-50205-x (PMC13369874; doi:10.1038/s41598-026-50205-x)
Supplement: Supplementary file 1 — Supplementary Material 1 [file 41598_2026_50205_MOESM1_ESM.docx]

Supplementary Files

**Threonine sulfation: a rare post translational modification in insect adipokinetic hormones**

Gerd Gäde^1#*^, Simone König^2#^, Sameer S. Kulkarni^3,4^, Richard J. Payne^3,4^, Heather G. Marco^1^

^1^ Department of Biological Sciences, University of Cape Town, Rondebosch, South Africa

^2^ Service Unit Proteomics, Medical Faculty, University of Münster, Münster, Germany

^3^ School of Chemistry, The University of Sydney, Sydney, NSW 2006, Australia

^4^ Australian Research Council Centre of Excellence for Innovations in Peptide and Protein Science, Sydney, NSW 2006, The University of Sydney, Australia

^#^ Equal contribution

*Corresponding author: gerd.gade@uct.ac.za

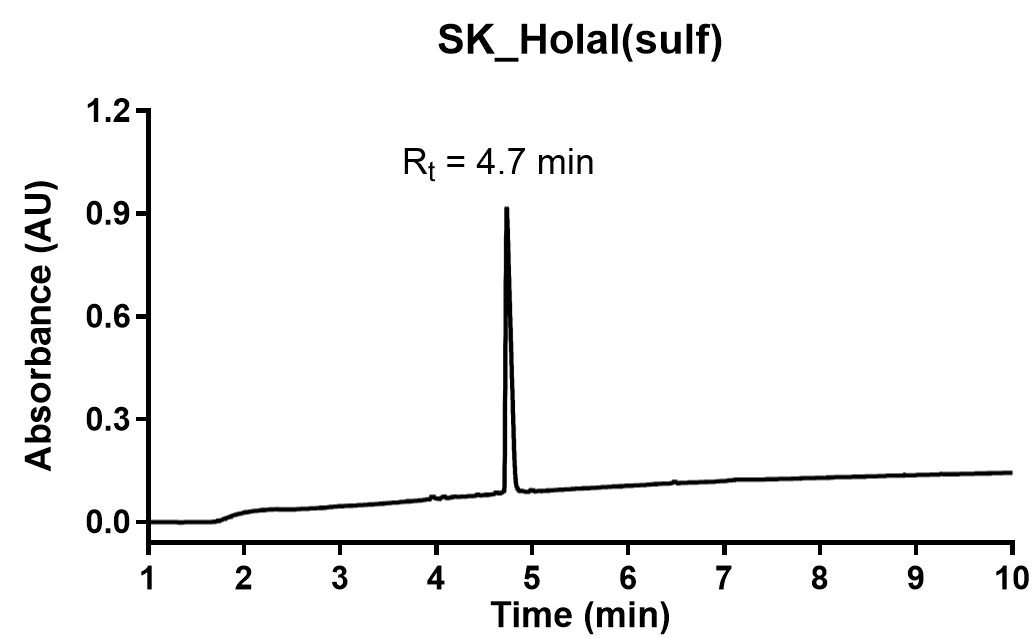

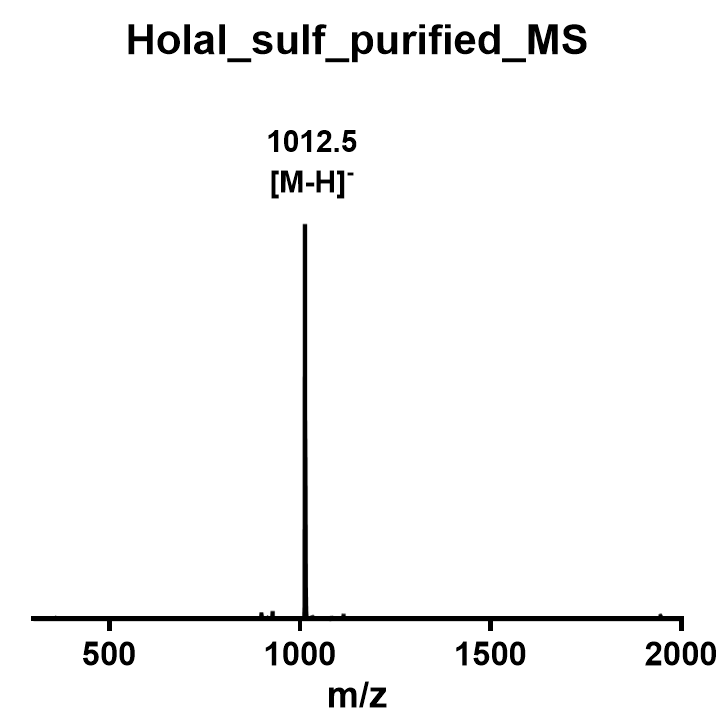


**B)**

**A)**

**Figure S1.** Structure control of purified synthetic sulfated Schgr-AKH-II. **(A)** Analytical UPLC trace using C18 column (1-90% B over 10 min, 0.1% formic acid). **(B)** ESI-MS relative abundance, calculated mass [M-H]^-^: 1012.4, mass found (ESI -ve) 1012.5. The overall yield of the synthesis was 10% (2.5 mg).

**B)**

**A)**


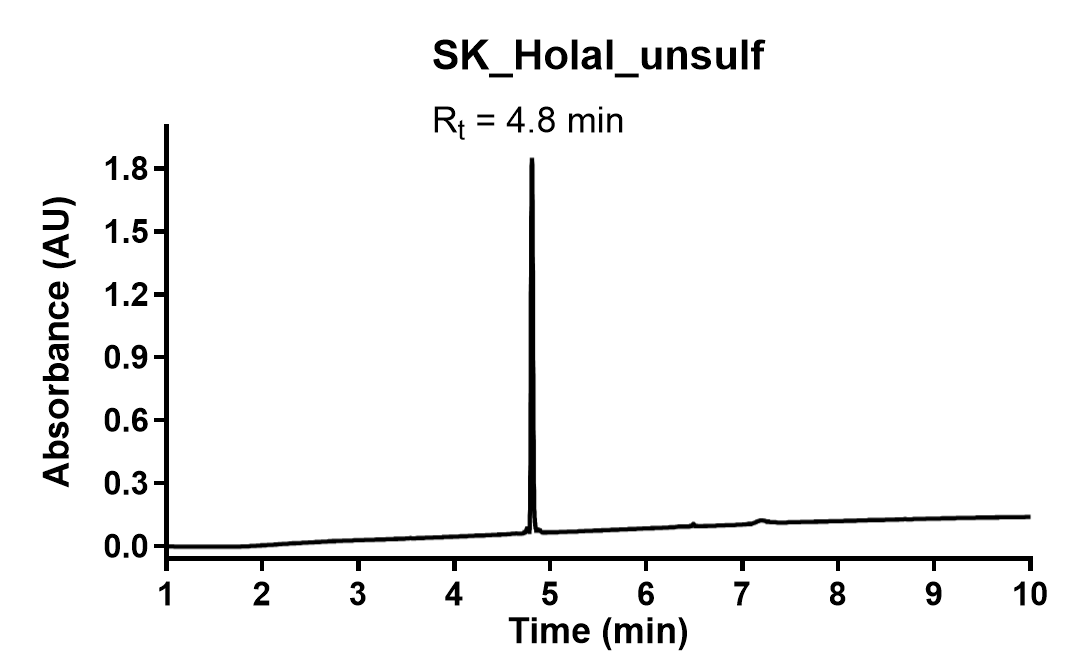


**Figure S2.** Structure control of purified synthetic Schgr-AKH-II. **(A)** Analytical UPLC trace using C18 column (1-90% B over 10 min, 0.1% TFA). **(B)** ESI-MS relative abundance. Calculated mass MH^+^ 934.4, mass found (ESI +ve) 934.8. Dimers were detected at *m/z* 1869.2. The overall yield of the synthesis was 60% (28 mg).

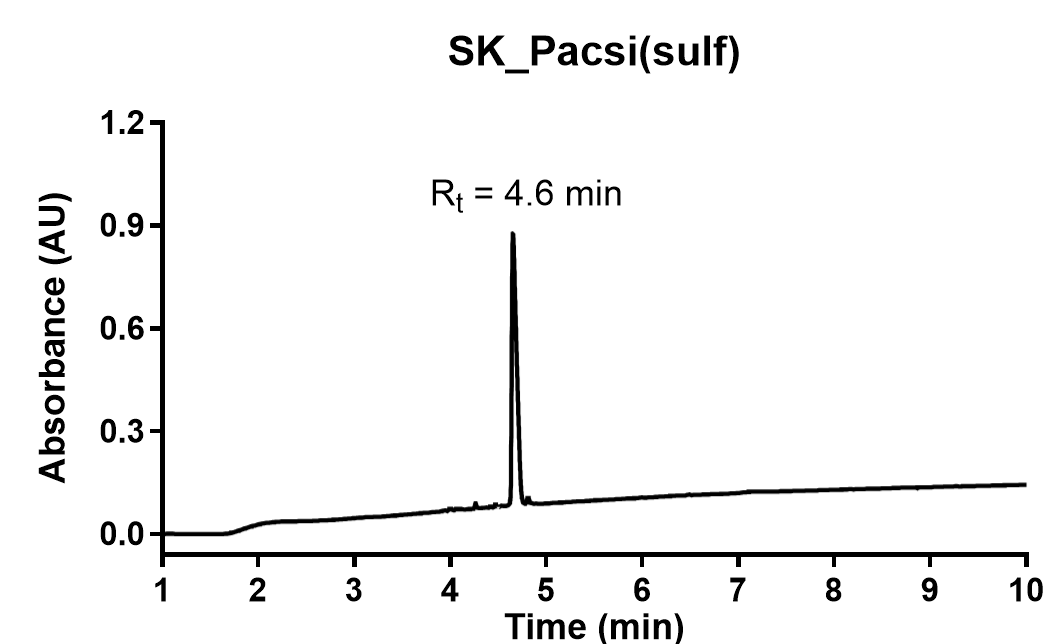

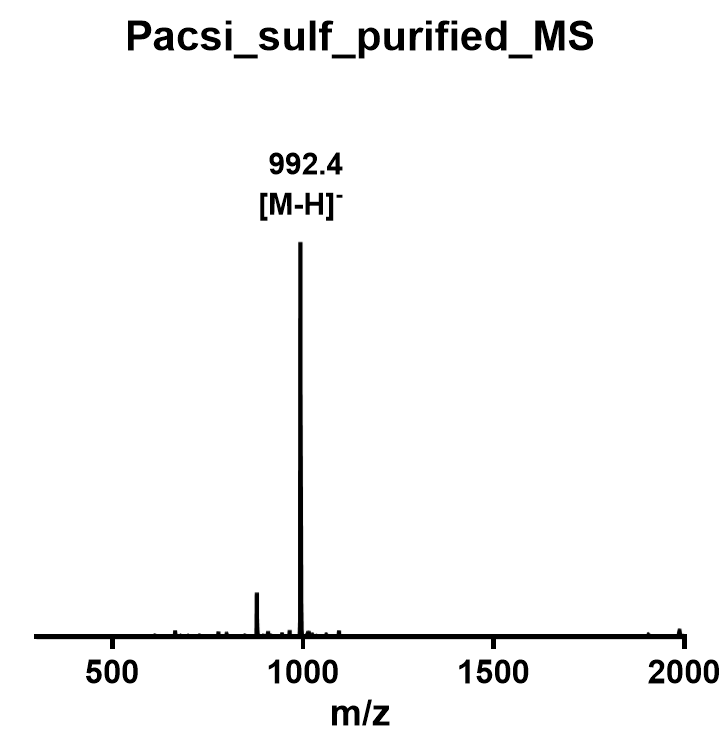


**B)**

**A)**

**Figure S3.** Structure control of purified synthetic sulfated Pacsi-AKH. **(A)** Analytical UPLC trace C18 column (1-90% B over 10 min, 0.1% formic acid). **(B)** ESI-MS relative abundance, calculated mass [M-H]^-^ 992.4, mass found (ESI -ve) 992.4. The overall yield of the synthesis was 8% (2.0 mg).

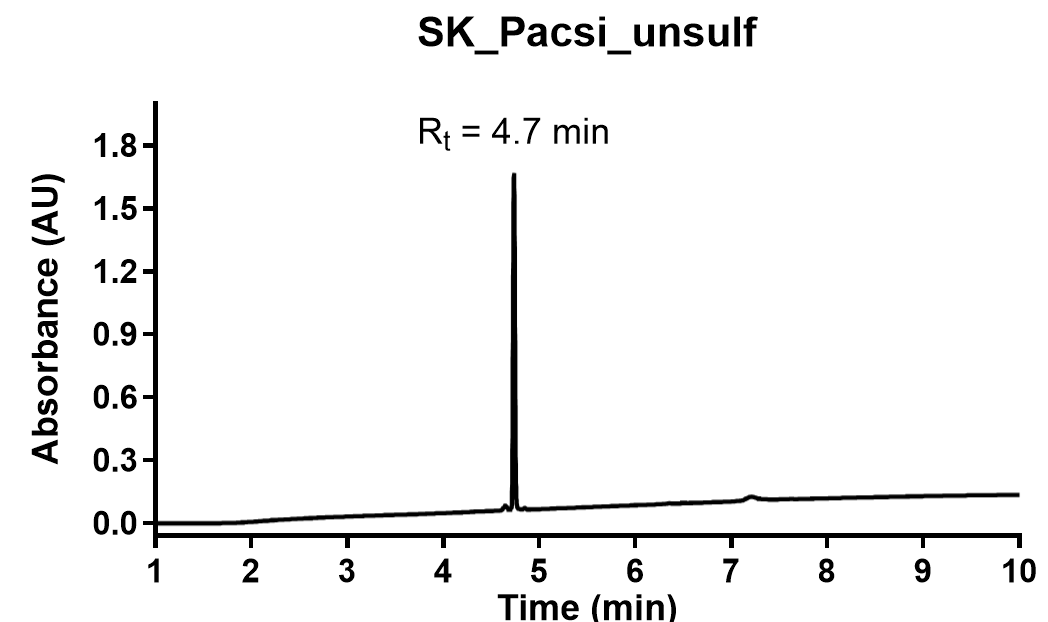


**A)**

**B)**

**Figure S4.** Structure control of purified synthetic Pacsi-AKH. **(A)** Analytical UPLC trace using C18 column (1-90% B over 10 min, 0.1% TFA). **(B)** ESI-MS relative abundance, calculated mass MH^+^ 914.5, mass found (ESI +ve) 914.9. Dimers were detected at *m/z* 1829.2. The overall yield of the synthesis was 53% (24 mg).

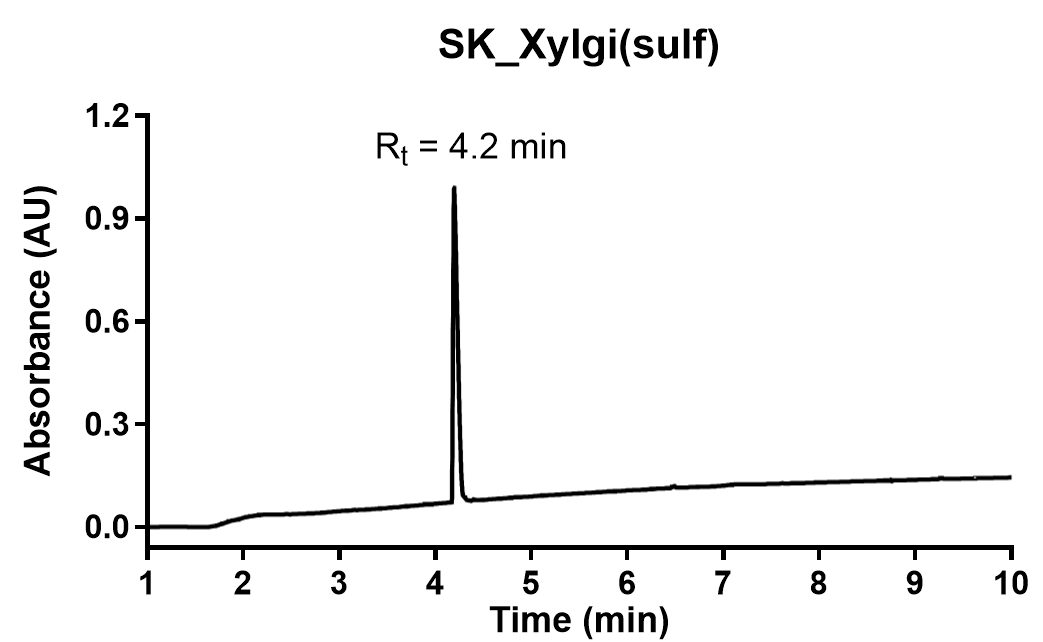

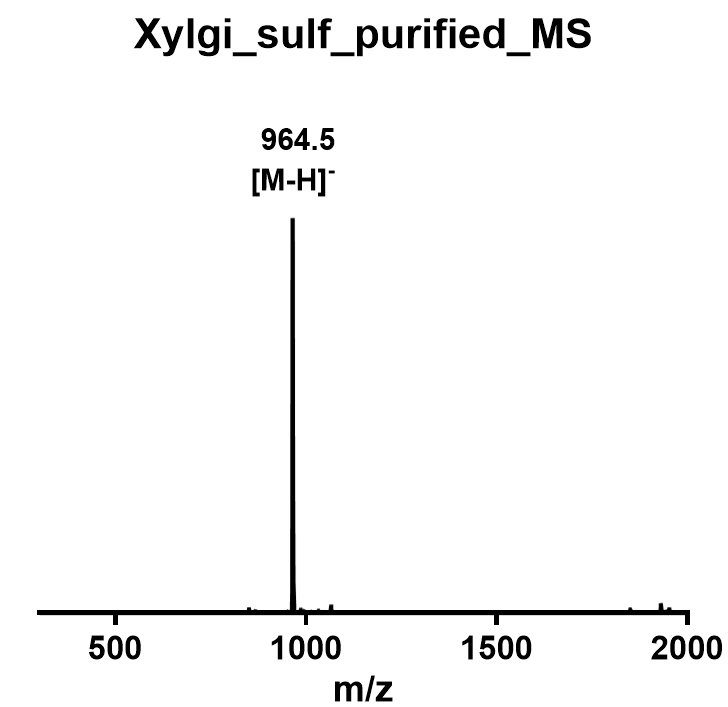


**A)**

**Figure S5.** Structure control of purified synthetic sulfated Penid-AKH. **(A)** Analytical UPLC trace using C18 column (1-90% B over 10 min, 0.1% formic acid). **(B)** ESI-MS relative abundance, calculated mass [M-H]^-^ 964.4, mass found (ESI -ve) 964.5. The overall yield of the synthesis was 3% (1.3 mg).

**B)**

**A)**


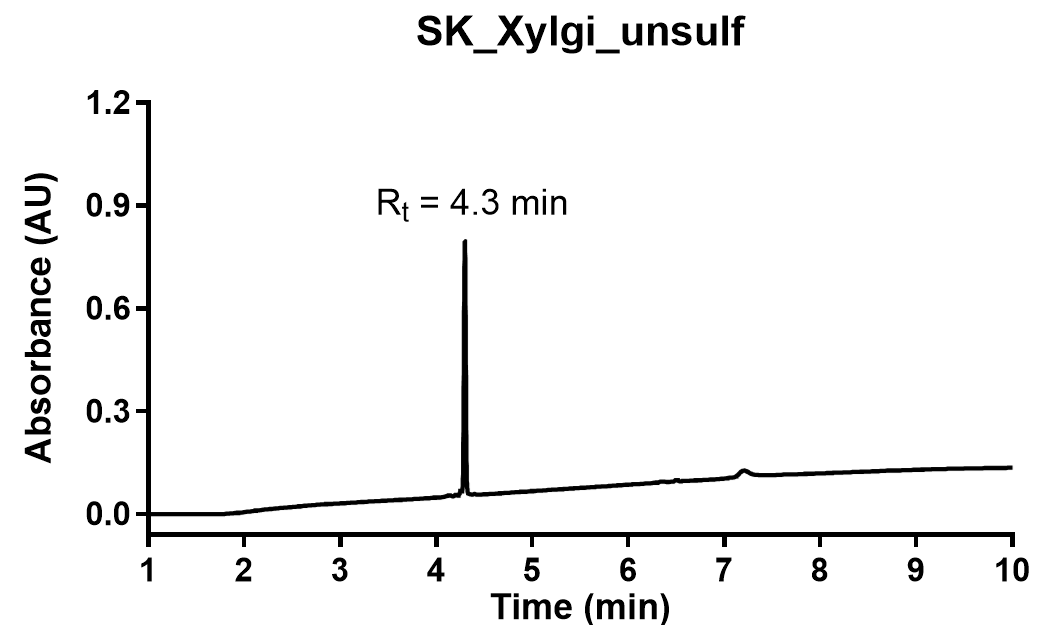


**Figure S6.** Structure control of purified synthetic Penid-AKH. **(A)** Analytical UPLC trace using C18 column (1-90% B over 10 min, 0.1% TFA). **(B)** ESI-MS relative abundance, calculated mass MH^+^ 886.4, mass found (ESI +ve) 886.5. Dimers were detected at *m/z* 1773.4. The overall yield of the synthesis was 43% (19 mg).


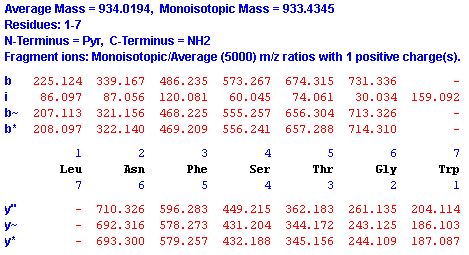


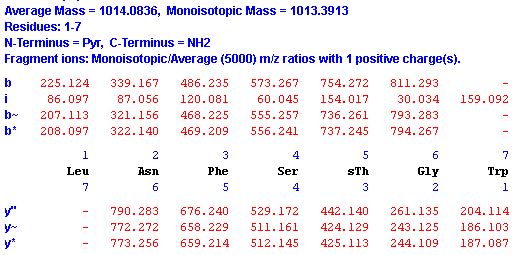


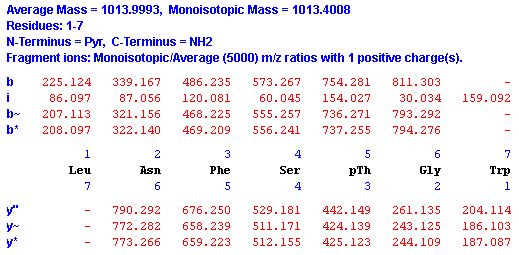


**Figure S7.** Expected fragment ions for Schgr-AKH-II and its sulfated and phosphorylated forms (sTh, pTh) as calculated by MassLynx software.


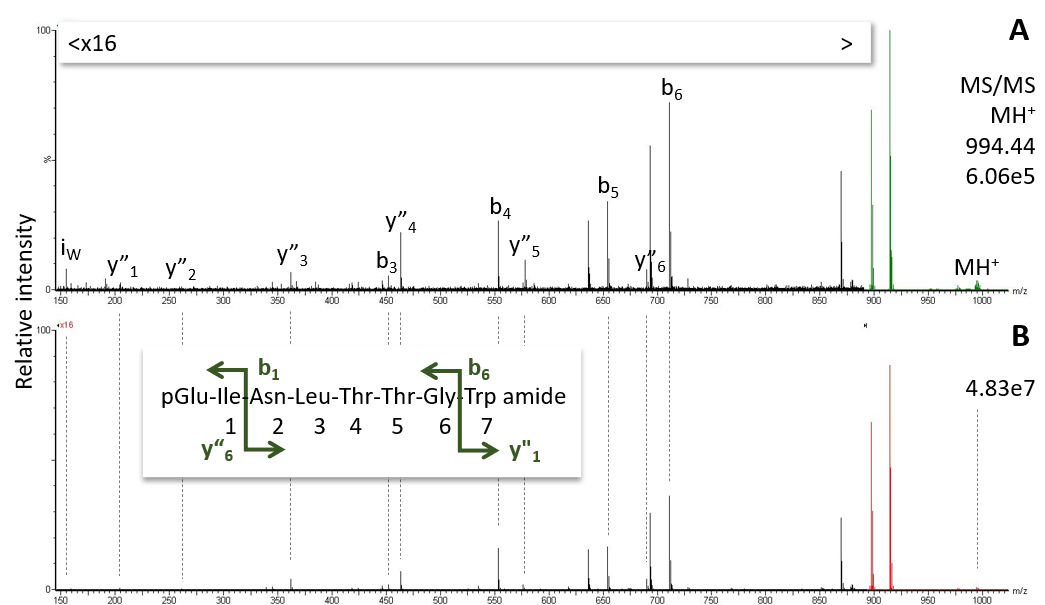


**Figure S8.** CID spectra of (A) the modified Pacsi-AKH detected in the CC extract of *D. derbyana* and (B) a synthetic sulfated peptide of the same sequence. The fragment ion spectra of both peptides matched. For theoretically expected ions, see Supplementary Fig. S9.


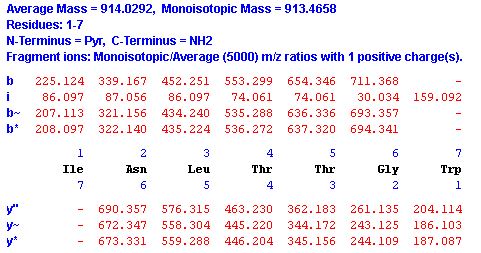


**Figure S9.** Expected fragment ions for Pacsi-AKH as calculated by MassLynx software.


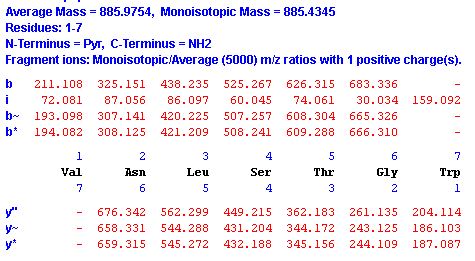


**Figure S10.** Expected fragment ions for Penid-AKH as calculated by MassLynx software.


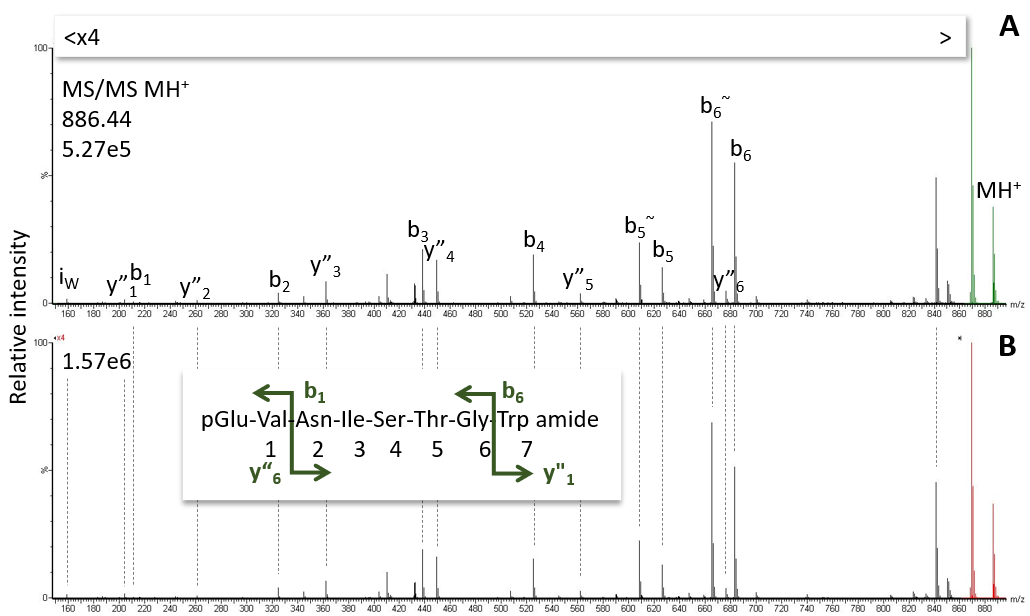


**Figure S11.** CID spectrum of a peptide detected in the CC extract of *X. gideon* (A) in comparison with the fragmentation of the peak for the unmodified synthetic Penid-AKH generated by in-source decay (B). The fragment ions of both peptides match. For expected ions, see Supplementary Fig. S10.


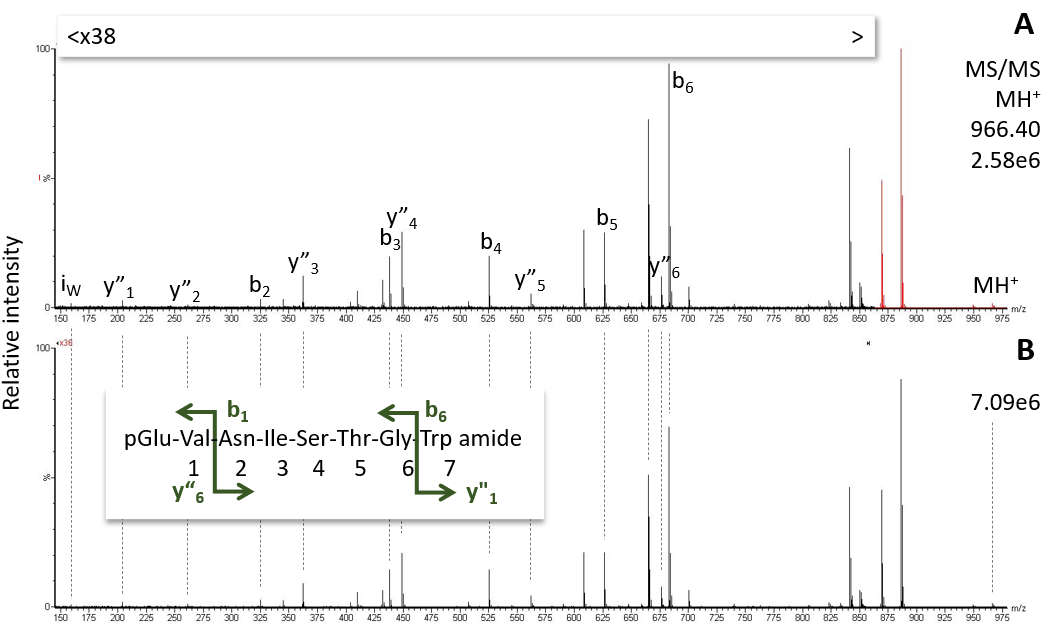


**Figure S12.** CID spectra of a sulfated Penid-AKH detected in the CC extract of *S. spurius* (A) in comparison with the synthetic peptide (B). The fragment ions of both peptides match. For expected ions, see Supplementary Fig. S10.
